# Supplementary material for: A Genome-Wide Association Study of Senegalese Sorghum Seedlings Responding to Pathotype 5 of Sporisorium reilianum
Source: Plants (Basel). 2022 Nov 7;11(21):2999. doi: 10.3390/plants11212999 (PMC9654544; doi:10.3390/plants11212999)
Supplement: Supplementary file 1 [file plants-11-02999-s001.zip › Supplementary Table S1.pdf]

**Supplementary Table S1. Spot appearance rate and average time of spot detection.**

| Accession | Spot appearance rate (%) | Average time (Days) of detection | Accession | Spot appearance rate (%) | Average time (Days) of detection |
|---|---|---|---|---|---|
| PI514279 (+) | 100 | 5.8 ± 0.1 | PI514342 | 53.3 | 3.8 ± 0.4 |
| PI514284 (+) | 100 | 4.3 ± 0.1 | PI514337 | 52.9 | 5.2 ± 0.1 |
| PI514287 | 100 | 4.4 ± 0.3 | PI514388 | 52.6 | 3.7 ± 0.2 |
| PI514289 | 100 | 3.6 ± 0.1 | PI514364 | 52.4 | 4.7 ± 0.1 |
| PI514295 | 100 | 4.5 ± 0.2 | PI514436 | 52.2 | 5.2 ± 0.1 |
| PI514306 | 100 | 4.3 ± 0.3 | PI514293 | 50 | 4.7 ± 0.4 |
| PI514308 | 100 | 5.4 ± 0.2 | PI514424 | 50 | 5.9 ± 0.1 |
| PI514309 | 100 | 3.9 ± 0.2 | PI514433 | 50 | 4.2 ± 0.9 |
| PI514311 | 100 | 3.4 ± 0.1 | PI514453 | 50 | 5.0 ± 0.4 |
| PI514325 | 100 | 3.8 ± 0.2 | PI514371 | 47.6 | 4.2 ± 0.3 |
| PI514326 | 100 | 4.0 ± 0.2 | PI514346 | 46.7 | 5.0 ± 0 |
| PI514333 | 100 | 3.7 ± 0.2 | PI514401 | 46.7 | 4.7 ± 0.2 |
| PI514345 | 100 | 4.4 ± 0.2 | PI514360 | 45.5 | 4.0 ± 0.1 |
| PI514394 | 100 | 4.8 ± 0.1 | PI514448 | 45.5 | 4.0 ± 0 |
| PI514412 | 100 | 4.6 ± 0.2 | PI514467 | 45 | 4.3 ± 0.4 |
| PI514449 | 100 | 4.2 ± 0.3 | PI514429 | 44.4 | 2.6 ± 0.2 |
| PI514478 | 100 | 4.0 ± 0.6 | PI514379 | 43.8 | 4.6 ± 0.2 |
| PI609251 | 100 | 4.0 ± 0 | PI514368 | 42.9 | 3.9 ± 0.4 |
| PI514294 | 95.7 | 4.4 ± 0.2 | PI514392 | 41.7 | 4.8 ± 0.4 |
| PI514380 | 95.7 | 3.9 ± 0.1 | PI514439 | 40 | 3.8 ± 0.4 |
| PI514341 | 95.5 | 4.3 ± 0.1 | PI514354 | 36.8 | 4.0 ± 0 |
| PI514316 | 95 | 4.3 ± 0.2 | PI514455 | 36.4 | 5.5 ± 0.3 |
| PI514340 | 95 | 5.1 ± 0.2 | PI514372 | 33.3 | 4.3 ± 0.2 |
| PI514283 | 94.1 | 4.4 ± 0.3 | PI514374 | 31.6 | 4.2 ± 0.2 |
| PI514324 | 93.8 | 4.2 ± 0.1 | PI514432 | 31.3 | 4.4 ± 0.2 |
| PI514301 | 92.9 | 4.5 ± 0.2 | PI514376 | 30 | 4.3 ± 0.3 |
| PI514334 | 92.9 | 4.1 ± 0.3 | PI514456 | 30 | 4.0 ± 0 |

| | | | | | |
|---|---|---|---|---|---|
| PI514344 | 91.7 | 4.5 ± 0.1 | PI514296 | 28.6 | 5.5 ± 0.2 |
| PI514299 | 91.3 | 4.0 ± 0.2 | PI514403 | 28.6 | 4.7 ± 0.7 |
| PI514313 | 90.9 | 4.0 ± 0.2 | PI514405 | 28.6 | 4.8 ± 0.8 |
| PI514317 | 90.9 | 4.0 ± 0.3 | SC748-5 | 28.6 | 4.0 ± 0 |
| PI514338 | 90.9 | 5.0 ± 0.1 | PI514322 | 27.3 | 5.0 ± 0.6 |
| PI514297 | 90 | 4.9 ± 0.3 | PI514425 | 27.3 | 4.3 ± 0.3 |
| PI514298 | 89.7 | 5.2 ± 0.2 | PI514434 | 27.3 | 4.7 ± 0.9 |
| PI514339 | 89.5 | 4.5± 0.1 | PI514395 | 26.7 | 5.0 ± 0 |
| PI514347 | 88.9 | 3.4 ± 0.3 | PI514400 | 23.5 | 4.8 ± 0.5 |
| PI514361 | 88 | 3.9 ± 0.2 | PI514428 | 23.5 | 2.8 ± 0.5 |
| PI514314 | 87.5 | 4.6 ± 0.2 | PI514452 | 23.1 | 4.0 ± 0 |
| PI514302 | 86.7 | 4.7 ± 0.1 | PI514465 | 23.1 | 4.3 ± 0.3 |
| PI514377 | 85.2 | 4.1 ± 0.2 | PI514399 | 21.1 | 4.0 ± 0 |
| PI514373 | 85 | 2.8 ± 0.1 | PI514336 | 20 | 4.5 ± 0.5 |
| PI514414 | 85 | 4.2 ± 0.2 | PI514382 | 20 | 4.3 ± 0.3 |
| PI514381 | 84.2 | 4.0 ± 0.1 | PI514396 | 20 | 5.5 ± 0.5 |
| PI514375 | 83.3 | 5.0 ± 0.3 | PI514404 | 20 | 5.5 ± 0.5 |
| PI514378 | 83.3 | 3.8 ± 0.3 | PI514438 | 20 | 5.5 ± 0.5 |
| PI514312 | 82.6 | 3.6 ± 0.2 | PI514454 | 20 | 5.0 ± 0 |
| PI514282 | 81.8 | 4.2 ± 0.3 | PI514457 | 20 | 6.0 ± 0 |
| PI514285 | 81.8 | 4.6 ± 0.2 | PI514461 | 20 | 5.0 ± 0 |
| PI514286 | 81.8 | 3.7 ± 0.4 | PI514473 | 20 | 3.0 ± 0 |
| PI514288 | 81.8 | 4.9 ± 0.2 | PI514335 | 18.2 | 5.0 ± 0 |
| PI514290 | 81.8 | 3.8 ± 0.3 | PI514387 | 18.2 | 5.0 ± 0 |
| PI514300 | 80 | 4.6 ± 0.4 | PI514444 | 18.2 | 3.5 ± 0.5 |
| PI514303 | 80 | 4.8 ± 0.3 | PI514440 | 16.7 | 6.0 ± 0 |
| PI514332 | 80 | 4.1 ± 0.2 | PI514418 | 14.3 | 4.5 ± 0.5 |
| PI514472 | 80 | 4.7 ± 0.3 | PI514391 | 13.3 | 4.5 ± 1.5 |
| PI514310 | 78.6 | 4.1 ± 0.3 | PI514423 | 13.3 | 4.0 ± 0 |
| PI514318 | 76.9 | 4.4 ± 0.2 | PI514458 | 12.5 | 3.5 ± 0.5 |

| | | | | | |
|---|---|---|---|---|---|
| PI514351 | 76.2 | 4.4 ± 0.2 | PI514383 | 10 | 4 |
| PI514362 | 76 | 4.3 ± 0.2 | PI514397 | 10 | 6 |
| PI514321 | 75 | 5.7 ± 0.2 | PI514427 | 10 | 2 |
| PI514419 | 72.7 | 3.5 ± 0.2 | PI514466 | 10 | 4 |
| PI514355 | 72.2 | 3.5 ± 0.2 | BTx643 | 10 | 4 |
| PI514291 | 71.9 | 4.2 ± 0.2 | PI514367 | 0 | - |
| PI514320 | 71.4 | 4.2 ± 0.4 | PI514390 | 0 | - |
| PI514352 | 71.4 | 3.7 ± 0.2 | PI514398 | 0 | - |
| PI514356 | 71.4 | 4.4 ± 0.2 | PI514409 | 0 | - |
| PI514304 | 70.8 | 4.8 ± 0.3 | PI514417 | 0 | - |
| PI514319 | 70.6 | 4.3 ± 0.3 | PI514430 | 0 | - |
| PI514323 | 70 | 4.3 ± 0.3 | PI514435 | 0 | - |
| PI514431 | 70 | 3.3 ± 0.2 | PI514459 | 0 | - |
| PI514437 | 70 | 4.2 ± 0.3 | PI514460 | 0 | - |
| PI514446 | 70 | 4.3 ± 0.4 | PI514462 | 0 | - |
| PI514353 | 68.8 | 4.2 ± 0.2 | PI514463 | 0 | - |
| PI514366 | 65 | 2.6 ± 0.1 | PI514464 | 0 | - |
| PI514363 | 64.7 | 4.2 ± 0.3 | PI514468 | 0 | - |
| PI514343 | 62.5 | 4.2 ± 0.2 | PI514469 | 0 | - |
| PI514349 | 61.9 | 4.5 ± 0.1 | PI514471 | 0 | - |
| PI514393 | 60 | 5.2 ± 0.2 | PI514474 | 0 | - |
| PI514292 | 58.3 | 4.0 ± 0.5 | PI514475 | 0 | - |
| PI514307 | 58.3 | 4.0 ± 0.2 | BTx623 | 0 | - |
| PI514411 | 58.3 | 4.9 ± 0.2 | BTx635 | 0 | - |
| PI514426 | 58.3 | 4.4 ± 0.4 | PI514279 Control (-) | 0 | - |
| PI514350 | 57.1 | 3.6 ± 0.4 | PI514282 Control (-) | 0 | - |
| PI514305 | 55.6 | 3.7 ± 0.5 | PI514284 Control (-) | 0 | - |
| PI514348 | 55.6 | 4.9 ± 0.3 | PI514303 Control (-) | 0 | - |

| | | | | | |
|---|---|---|---|---|---|
| PI514280 | 54.5 | 4.3 ± 0.3 | TAM428 | 0 | - |
| PI514420 | 53.8 | 4.6 ± 0.4 | Pearson's correlation = -0.12 with *p*-value = 0.14 | | |

Among 163 Senegalese accessions, 146 accessions showed symptoms after inoculation with P5 of *S. reilianum*, but the infection rate based on spot appearance varied. Mean values (days) and standard error means (SEM) are shown for timing of symptom confirmation. Accessions in Table 1 are listed from the highest to the lowest infection rates.
